# The Evolving Role of Nuclear Medicine and Molecular Imaging: Theranostics and Personalized Therapeutic Applications

**DOI:** 10.4274/mirt.30502

**Published:** 2018-02-01

**Authors:** M. Fani Bozkurt, Zehra Özcan

**Affiliations:** 1 Hacettepe University Medical School, Department of Nuclear Medicine, Ankara, Turkey; 2 Ege University Medical School, Department of Nuclear Medicine, İzmir, Turkey

## OVERVIEW

During the last decade, there have been excellent and very rapid advances in “Nuclear Medicine and Molecular Imaging” throughout the world. The developments in radiopharmaceuticals induced evolution of nuclear medicine from imaging certain biologic features to targeted drug delivery designed for the specific characteristics of an individual patient’s disease. While the use of therapeutic radioisotopes was an important but minor component of the therapeutic oncology in the past, now with the development of “Theranostic” applications, intelligent options for targeted internal radionuclide treatments became possible in a variety of tumors and Theranostics started to give rise to a paradigm shift in oncology.

“Theranostic” concept in nuclear medicine represent both diagnostic and therapeutic function in one drug formulation and while bridging these two goals. The term “Theranostic” is generated from ‘therapy’ and ‘diagnostics/diagnosis’ (1). Actually Iodine-131 is the oldest and the most common isotope in theranostic applications. In this case, the same radioisotope Iodine-131 serves for both diagnostic and therapeutic purpose on the basis of using the same target, although Iodine-123 which is the pure gamma emitter isotope of Iodine can take part as the diagnostic agent. Theranostic approach also includes the use of different radioistopes but again depending on the principle of using the same target for both diagnosis and therapy Recently, there have been new “theranostics” agents in clinical practice, which are good examples for theranostic approach with two different radioisotopes. For instance, somatostatin receptors on the surface of the neuroendocrine neoplasia have been used as targets for radionuclide imaging and treatment on the basis of “theranostic” approach. PET Imaging with positron emitter Ga-68 labelled peptides which show affinity to somatostatin receptors and treatment with beta emitter Y-90/Lu-177 labelled peptides targeting these receptors gained wide acceptance in the field (2,3).

There has been a growing interest also for the use theranostic approach in prostate cancer which affects a great number of males. The presence of prostate specific membrane antigen (PSMA) expression in prostate cancer served as a basis for the idea of targeting these receptors for PET imaging using Ga-68 labelled PSMA and consequently treating with Lu-177 labelled PSMA (4,5). The potential for drug delivery system using theranostic basis also enables us to administer therapy according to the individual requirements of the patient. As the tumor nature is heterogenous, a specific drug indicating a certain characteristic will be a therapeutic option only for a subset of tumors. So the treatment will be customized for only patients whose tumor contains very specific proteins or receptors, which will eventually result with a more “precise” therapy.

Recently, Radium 223 which is a calcium mimicking radioisotope has been introduced as an effective treatment in metastatic castration resistant prostate cancer patients with bone involvement only (6). While delivering alpha emission to the metastatic deposits detected by bone imaging tracers, Ra-223 provides improvement in patient survival and skeletal related events. Therefore, Ra-233 treatment became available in all over the world and covered in most European countries (7). Being recently licensed in Turkey, it is believed that this new targeted radionuclide treatment will also be available in our patients and alpha emitting radionuclides will open a new era in therapeutic nuclear medicine.

It is clear that nuclear medicine and molecular imaging will enlarge its role in the early diagnosis and treatment of cancer and also will be a driving force in personalized medicine using theranostic concepts. Finally, while completing a successful year and starting a new year, we, the editors of MIRT, hope that scientific researches in our field will expand more and MIRT will be a leading publication for all these new ideas and researches promoting diagnostic and therapeutic Nuclear Medicine applications.
